# Neural crest cell-placodal neuron interactions are mediated by Cadherin-7 and N-cadherin during early chick trigeminal ganglion assembly

**DOI:** 10.12688/f1000research.122686.2

**Published:** 2022-12-12

**Authors:** Caroline A. Halmi, Chyong-Yi Wu, Lisa A. Taneyhill

**Affiliations:** 1 Department of Animal and Avian Sciences, University of Maryland, College Park, Maryland, 20742, USA; 2 U.S. Food and Drug Administration, Silver Spring, Maryland, 20993, USA

**Keywords:** cadherins, neural crest cells, placode cells, trigeminal ganglion, chick embryo

## Abstract

**Background:** Arising at distinct positions in the head, the cranial ganglia are crucial for integrating various sensory inputs. The largest of these ganglia is the trigeminal ganglion, which relays pain, touch and temperature information through its three primary nerve branches to the central nervous system. The trigeminal ganglion and its nerves are composed of derivatives of two critical embryonic cell types, neural crest cells and placode cells, that migrate from different anatomical locations, coalesce together, and differentiate to form trigeminal sensory neurons and supporting glia. While the dual cellular origin of the trigeminal ganglion has been known for over 60 years, molecules expressed by neural crest cells and placode cells that regulate initial ganglion assembly remain obscure. Prior studies revealed the importance of cell surface cadherin proteins during early trigeminal gangliogenesis, with Cadherin-7 and neural cadherin (N-cadherin) expressed in neural crest cells and placode cells, respectively. Although cadherins typically interact in a homophilic ( *i.e.*, like) fashion, the presence of different cadherins expressed in neural crest cells and placode cells raises the question as to whether heterophilic cadherin interactions may also be occurring. Given this, the aim of the study was to understand whether Cadherin-7 and N-cadherin were interacting during initial trigeminal ganglion formation.

**Methods:** To assess potential interactions between Cadherin-7 and N-cadherin, we used biochemistry and innovative imaging assays conducted *in vitro* and *in vivo*, including in the forming chick trigeminal ganglion.

**Results: **Our data revealed a physical interaction between Cadherin-7 and N-cadherin.

**Conclusions:** These studies identify a new molecular basis by which neural crest cells and placode cells can aggregate *in vivo* to build the trigeminal ganglion during embryogenesis.

## Introduction

Cranial ganglia are sensory structures of the peripheral nervous system possessing the cell bodies of the cranial nerves. These ganglia and their associated nerves function in olfaction, taste, hearing, vision, and somatosensation. ^1^^–^^3^ The trigeminal ganglion contains three sensory branches (ophthalmic, maxillary, and mandibular) that innervate different regions of the face to mediate sensations of pain, touch, and temperature. ^3^^–^^5^ During embryonic development, two distinct cell populations, neural crest cells and neurogenic placode cells, intermingle and aggregate to generate the trigeminal ganglion. ^6^^–^^10^ These interactions have been studied for over 60 years and reveal that each cell type contributes distinctly to trigeminal ganglion formation, with neural crest cells acting as a scaffold for the integration of placode cell-derived neurons, while placodal neurons aid in the condensation of neural crest cells. ^7^^,^^10^^,^^11^ Moreover, ablation of either of these cell populations leads to severe defects in trigeminal ganglion development, indicating a reciprocal relationship. ^7^^,^^10^^,^^12^

Prior studies indicate that intercellular interactions during trigeminal ganglion formation are mediated, in part, by cadherin-based adhesion. Two cadherins, Cadherin-7 and neural cadherin (N-cadherin), are expressed in neural crest cells and placode cells, respectively, during chick trigeminal gangliogenesis. Expression of *Cadherin-7*, a type II classical cadherin, was discovered in migratory cranial neural crest cells in the chick embryo over 25 years ago. ^13^ More recent studies of Cadherin-7 protein confirmed previous *in situ* hybridization findings and noted Cadherin-7 in chick migratory cranial neural crest cells contributing to the trigeminal ganglion. ^14^ Both depletion and overexpression of Cadherin-7 impact the distribution of embryonic neural crest cells and placodal neurons, and as such, the overall morphology of the ganglion. N-cadherin, a type I classical cadherin, is present throughout development and has been found in derivatives of the endoderm, mesoderm, and ectoderm. ^15^ Notably, both ectodermal placode cells and their neuronal derivatives express N-cadherin ^16^ in the chick trigeminal ganglion. Knockdown of N-cadherin does not affect initial placode cell ingression and delamination from the ectoderm, ^16^ but leads to increased placodal neuron dispersal during trigeminal gangliogenesis. Conversely, N-cadherin overexpression causes aberrant aggregation of placodal neurons. ^16^ Modulation of N-cadherin levels appears to involve, in part, post-translational mechanisms linked to Slit1-Robo2 signaling in the developing chick trigeminal ganglion, ^16^ but specific details underlying this process are not known.

While the ability of cadherins to make homophilic interactions is well understood, cadherins can also make heterophilic ( *i.e.*, non-like) connections with other cadherins, either in the same (homotypic) or different (heterotypic) cell types. Observations of heterophilic cadherin interactions have been reported during normal development of the endoderm, ^17^ in establishing synaptic potentials within the hippocampus, ^18^ and during *Xenopus* gastrulation, ^19^ and are also noted in diseases such as cancer. ^20^ In addition, the atypical cadherins Fat and Dachsous are capable of forming heterodimers between neighboring homotypic cells. ^21^ Collectively, these results support the notion that heterophilic interactions can occur between different types of cadherins during development. While previous studies noted the formation of aggregates from mixtures of N-cadherin- and Cadherin-7-expressing cells *in vitro*, ^13^ the potential role of heterophilic cadherin interactions between neural crest cells and placode cell-derived neurons as they assemble the trigeminal ganglion has yet to be explored.

To address this question, we performed experiments to elucidate potential heterophilic interactions between Cadherin-7 and N-cadherin during the formation of the chick trigeminal ganglion. Our *in vivo* and *in vit*ro biochemistry and imaging data indicate Cadherin-7 and N-cadherin physically interact during trigeminal ganglion assembly and that this involves heterophilic interactions between Cadherin-7, expressed in neural crest cells, and N-cadherin, found in placodal neurons. These findings further clarify the reciprocal relationship observed between coalescing neural crest cells and placodal neurons during trigeminal gangliogenesis, providing an additional molecular basis for this process.

## Methods

### Chick embryos

Fertilized chicken eggs ( *Gallus gallus*) were obtained from the Department of Animal and Avian Sciences, University of Maryland, and Moyer’s Chicks, Inc. (PA), and incubated at 37°C in humidified incubators ( EggCartons.com, Manchaug, MA, USA). Embryos were staged by the Hamburger-Hamilton (HH) staging method ^22^ or by counting the number of somite pairs (somite stage, ss).

### Ethical approval

No ethical approval was required for this study for the chick embryos. The NIH Office for Protection from Research Risks has interpreted “live vertebrate animal” to apply to avians ( *e.g.*, chick embryos) only after hatching. Since our work does not utilize hatched chicks, no Institutional Animal Care and Use protocol for this work is necessary.

### Green fluorescent protein (GFP) reconstitution across synaptic partners (GRASP) cadherin construct preparation

Four different GRASP constructs were synthesized by GenScript (RRID:SCR_002891) to allow for incorporation of split GFP moieties (subunits 1-10 or subunit 11) into the extracellular domain of Cadherin-7 and N-cadherin, with the design based on similar plasmids generated in ^23^ and available in Addgene (m-sGFP1-10::NLG1 ( Addgene plasmid #44967; RRID:Addgene_44967) and m-sGFP11::NXN were gifts from Joshua Sanes ( Addgene plasmid #44968; RRID:Addgene_44968)). Briefly, each plasmid from Addgene was modified to remove the respective insert (either NLG1 or NXN), and, in its place, we inserted the *Cadherin-7* or *N-cadherin* cDNA sequence corresponding to the mature peptide. Sequence accuracy of constructs was confirmed by GenScript and expression of each cadherin was validated through immunocytochemistry.

### Cell culture and transfection assays

Chinese hamster ovary (CHO) cells (ATCC Cat# CCL-61, RRID:CVCL_0214; American Type Culture Collection) were cultured in Ham’s F12 media (10-080, Corning/Cellgro) supplemented with 10% fetal bovine serum (Genesee Scientific Cat#25-514H). Transient transfection assays were carried out using the Lipofectamine 2000 reagent (Thermo Fisher Scientific, Inc., Cat#11668019). Cells were grown to 90% confluency, and transfections were performed according to the manufacturer’s instructions and according to the protocols outlined in. ^24^^,^^25^ The chick *N-cadherin*-expressing (pCIG-N-cadherin) and empty (pCIG) vectors were gifts from Dr. Marianne Bronner (California Institute of Technology).

### Immunoprecipitations

Embryonic trigeminal ganglia were used for immunoprecipitations, with tissue harvested as described previously by. ^14^ ^,^ ^25^ ^,^ ^26^ Briefly, forming trigeminal ganglia were dissected, pooled, pelleted, flash-frozen in liquid nitrogen, and stored at -80°C. Cultured cells were scraped into 1X Phosphate-buffered Saline (1X PBS), pelleted, flash-frozen in liquid nitrogen, and stored at -80°C. Pellets were thawed on ice and lysed in lysis buffer (50 mM Tris pH 8.0, 150 mM NaCl, 1% IGEPAL CA-630) supplemented with cOmplete protease inhibitor cocktail tablets (Roche, Cat#04693124001) and 1 mM PMSF (Sigma Aldrich Cat#10837091001) for 30 minutes at 4°C with periodic mixing. Soluble fractions were collected following centrifugation at maximum speed for 15 minutes at 4°C (Microfuge 20R Centrifuge, Beckman Coulter, Inc., Cat#B31612), and protein concentration was quantified (BioPhotometer, Eppendorf, Cat#6131 26936) by Bradford assay (Thermo Fisher Scientific, Inc., Cat#1863028). Immunoprecipitations were carried out using protein A/G magnetic beads (Thermo Fisher Scientific, Inc., Cat#88802) according to the manufacturer’s instructions (Thermo Fisher Scientific, Inc.). Equivalent amounts of protein lysates (~120 μg) were incubated with 10 μg rabbit polyclonal N-cadherin antibody (Abcam Cat#ab12221, RRID:AB_298943) or normal rabbit IgG control (R&D Systems Cat#AB-105-C, RRID:AB_354266) overnight at 4°C with constant rotation. The following day, 0.25 mg washed protein A/G magnetic beads were incubated with the lysate/antibody mixture for one hour at room temperature with mixing. Following incubation, the samples were washed, equivalent volumes of SDS sample buffer were added, mixtures were boiled at 100°C for 10 minutes, magnetic beads were collected, and samples were loaded for immunoblotting as described below. Input amounts represent 5% (trigeminal ganglia) and 10% (cell culture) of the initial lysate amount used in the immunoprecipitation. Assays were conducted twice.

### Immunoblotting

Immunoblotting after immunoprecipitation was performed according to the protocol by Refs. 14, 25, 26. Samples were processed *via* SDS-PAGE (10% Mini-Protean TGX gel, BioRad #456-1034) in 1X Running Buffer (25 mM Tris (Thermo Fisher Scientific, Inc., Cat#BP-152-1), 192 mM glycine (Thermo Fisher Scientific, Inc., Cat#AC120070010), 0.1% sodium dodecyl sulfate (VWR, Cat#4095-02)) and then transferred to 0.45 μm BioTrace nitrocellulose membrane (Pall, Cat#66485) *via* wet transfer (Biorad, Mini-PROTEAN Tetra Vertical Cell for Mini Precast gels, Cat#1658004) in 1X Transfer Buffer (Running Buffer + 10% Methanol (Thermo Fisher Scientific, Inc., Cat#A452-4)) according to the manufacturer’s guidelines. For immunoblotting, membranes were blocked in blocking buffer (1X PBS + 0.1% Tween-20 (Sigma Aldrich, Cat#P1379-500ML)) (PTW) + 5% non-fat milk (Carnation Instant Nonfat Dry Milk). Next, primary antibodies against mouse monoclonal Cadherin-7 (1:150, DSHB, Cat#ccd7-1, RRID:AB_528111) or rabbit polyclonal N-cadherin (1:1000, Abcam Cat#ab12221) were diluted as indicated in blocking buffer and incubated overnight with shaking at 4°C. Unbound primary antibodies were washed off with PTW (three times, 10 minutes each), followed by incubation at room temperature for 45 minutes with the following secondary antibodies diluted in blocking buffer (1:10,000): goat anti-mouse polyclonal IgG (H&L) antibody peroxidase conjugated (Rockland Cat# 610-1302, RRID:AB_219656) or goat anti-rabbit polyclonal IgG (H&L) secondary antibody peroxidase conjugated (Rockland Cat# 611-1302, RRID:AB_219720). After washing three times, 10 minutes each, in PTW, proteins were detected using enhanced chemiluminescent substrates mixed in a 1:1 ratio (SuperSignal West Pico PLUS Chemiluminescent Substrate (Thermo Fisher Scientific, Inc., Cat#34580) or SuperSignal West Femto Maximum Sensitivity Substrate (Thermo Fisher Scientific, Inc., Cat#34095)). Immunoblot images for figures were gamma-modified and processed using Adobe Photoshop (RRID:SCR_014199) CC 2019 (20.0.6 release, Adobe Systems, San Jose, CA, USA).

### Immunostaining

Embryos collected at various stages, or cultured cells in two-well chamber slides (LAB-TEK, Cat#154461), were used for immunostaining. For the former, detection of various proteins was performed on 14 μm transverse sections following 4% paraformaldehyde (PFA) fixation overnight, gelatin embedding, and cryostat sectioning, according to the protocol described previously by. ^14^^,^^26^^,^^27^ For the latter, cells were fixed in 4% PFA for 15 minutes, followed by immunocytochemistry. Tissue or cells were permeabilized by washing two times, 10 minutes each, in 1X PBS + 0.1% Triton X-100 (Sigma Aldrich, Cat#TX1568-1) (PBSTX), followed by a one-hour blocking step of PBSTX + 10% sheep serum (Sigma Aldrich, Cat#S2263-100ML). All primary and secondary antibodies were diluted in 1X PBSTX + 5% sheep serum. The following antibodies and dilutions were used for immunostaining: mouse monoclonal anti-Cadherin-7 (1:50-1:70, DSHB Cat#ccd7-1); rat monoclonal anti-N-cadherin (1:50, DSHB Cat#MNCD2, RRID:AB_528119); mouse monoclonal anti-human natural killer-1 (HNK-1) (1:100, DSHB Cat#3H5, RRID:AB_2314644); and mouse monoclonal anti-Tubulin beta-3 chain (Tubb3) (1:500, Abcam Cat# ab78078, RRID:AB_2256751). The following secondary antibodies were used at 1:200-1:500 dilutions: goat anti-mouse polyclonal IgG (H + L) Cross-Adsorbed Secondary Antibody, Alexa Fluor 594 (Thermo Fisher Scientific Cat# A-11005, RRID:AB_2534073) and goat anti-mouse polyclonal IgG (H + L) Cross-Adsorbed Secondary Antibody, Alexa Fluor 647 (Thermo Fisher Scientific Cat# A-21235, RRID:AB_2535804) (for Cadherin-7); goat anti-rat polyclonal IgG (H + L) Cross-Adsorbed Secondary Antibody, Alexa Fluor 594 (Thermo Fisher Scientific Cat# A-11007, RRID:AB_10561522) (for N-cadherin); goat anti-mouse polyclonal IgM (Heavy Chain) Secondary Antibody (Thermo Fisher Scientific Cat# A-21238, RRID:AB_2535807) (for HNK-1); and goat anti-mouse polyclonal IgG _2a_ Human ads-AF555 (SouthernBiotech Cat# 1080-32, RRID:AB_2794491) (for Tubb3). Sections were stained with 4′,6-diamidino-2-phenylindole (DAPI) to mark cell nuclei using DAPI-containing mounting media (DAPI Fluoromount-G, Southern Biotech, Cat#0100-20).

### *In ovo *electroporation

For sequential electroporation of both premigratory neural crest cells and trigeminal placode cells, unilateral chick neural tube electroporation to target neural crest cells contributing to the trigeminal ganglion was first performed, as described previously by. ^14^^,^^27^ Briefly, GRASP constructs were introduced unilaterally into premigratory midbrain neural crest cells in developing 3 to 5 somite stage (3-5ss) chick embryos at a concentration of 2.0-2.5 μg/μl, using fine glass needles to fill the chick neural tube. Platinum electrodes were placed on either side of the embryo, and two 25 V, 25 ms electric pulses were applied across the embryo. Once embryos reached HH10-11 (10-13ss), a unilateral ectodermal electroporation was carried out (on the same side of the embryo that was electroporated previously) to target trigeminal placode cells. ^26^ Electrodes were placed vertically on top of and below the embryo and three, 9 V pulses were delivered over 50 ms at 200 ms intervals. After electroporation, eggs were re-sealed with tape and parafilm and re-incubated for the desired time period (approximately 36 hours to reach HH15-16) prior to harvesting for fixation and transverse sectioning, which was carried out according to the protocol by. ^14^ Unilateral co-electroporation of N-cadherin split GFP constructs into trigeminal placode cells was carried out as described. ^26^

### Confocal imaging

For all experiments, images of at least five serial transverse sections through a minimum of three embryos, or eight cell culture images/replicates, were acquired with the LSM Zeiss 800 confocal microscope with Airyscan detection (Carl Zeiss Microscopy, Thornwood, NY, USA) at 20X magnification. Laser power, gain, and offset were kept consistent for the different channels during all experiments where possible. ZEN Digital Imaging for Light Microscopy (RRID:SCR_013672), version 2.3 software (Carl Zeiss Microscopy) and Adobe Photoshop CC 2019 (20.0.6 release) were used for image processing. For the *in vivo* GRASP experiments, images were false-colored in Adobe Photoshop to allow for better visualization and comparison of signal. Equivalent functions for image processing can be performed on Fiji (RRID:SCR_002285), which is freely available.

### Quantification

Images from both in vitro and in vivo experiments were analyzed using the Squassh ^28^ plugin from the MOSAICsuite in FIJI. ^29^ Segmentation parameters were adjusted per experiment, to minimize interfering background signal. For the *in vitro* assays, the amount of GFP positive and DAPI positive cells were counted and divided by each other to determine transfection efficiency. For N-cadherin *in vivo* control experiments, GFP positive cells and N-cadherin positive cells were counted and divided by each other to determine electroporation efficiency. For the *in vivo* heterophilic cadherin experiments, the number of GFP regions were counted per section, and averaged to find the average amount of GFP-positive regions. After batch analysis, the quality of image-segmentation was confirmed visually in order to ensure accurate segmentation.

## Results

### Heterophilic cadherin interactions exist in the forming chick trigeminal ganglion

**Figure 1.**
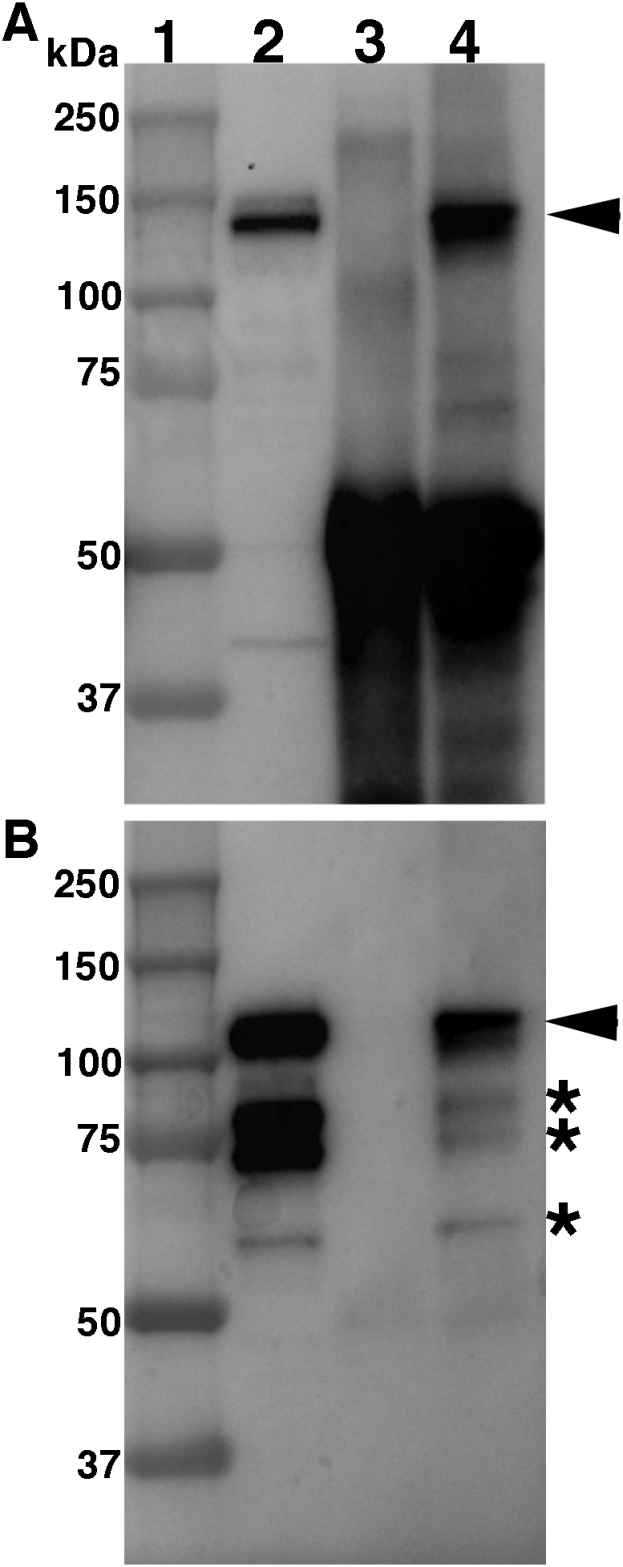
Cadherin-7 and N-cadherin physically interact *in vivo.* Lysate from HH15-16 trigeminal ganglion tissue was incubated with either an antibody against N-cadherin or with whole rabbit IgG serum as a control. Immunoprecipitated proteins were captured with protein A/G beads, separated by SDS-PAGE, followed by immunoblotting for N-cadherin (A) and Cadherin-7 (B). Lanes 1-4 are as follows: 1) Protein ladder; 2) Input, trigeminal ganglia lysate; 3) trigeminal ganglia lysate following IP with rabbit IgG; and 4) trigeminal ganglia lysate following IP with N-cadherin antibody. Arrowheads point to N-cadherin (A) or Cadherin-7 (B), respectively, while asterisks identify Cadherin-7 immunoreactive products as observed previously. ^14^ N=2 experiments. N-cadherin, neural cadherin; IP, immunoprecipitation.

Cranial neural crest cells and trigeminal placodal neurons express distinct cadherins, Cadherin-7 and N-cadherin, respectively, during early trigeminal gangliogenesis. ^14^^,^^16^ Given these findings, we sought to determine whether these specific cadherins facilitated trigeminal ganglion assembly through heterophilic interactions. To address this, we performed co-immunoprecipitation assays on lysates prepared from dissected forming trigeminal ganglia of HH15-16 chick embryos ( Figure 1). ^47^

Analysis of immunoprecipitated proteins by SDS-PAGE followed by immunoblotting for N-cadherin revealed detection of N-cadherin within the forming trigeminal ganglia ( Figure 1A, lane 2) and in N-cadherin immunoprecipitates with this antibody ( Figure 1A, lane 4), but not with the rabbit IgG serum ( Figure 1A, lane 3). These data indicate that the N-cadherin antibody can effectively immunoprecipitate N-cadherin from trigeminal ganglia tissue, providing us with a key experimental tool to identify other proteins that physically interact with N-cadherin *in vivo.*

To this end, we next performed immunoblotting using a validated Cadherin-7 antibody ^13^^,^^14^ ( Figure 1B). Our data again reveal a band corresponding to Cadherin-7 observed in the trigeminal ganglia lysate input sample ( Figure 1B, lane 1, arrowhead), along with immunoreactive lower molecular weight bands ( Figure 1B, asterisk, *) containing portion(s) of the Cadherin-7 extracellular domain, as observed in our prior work. ^14^ Strikingly, we also observed Cadherin-7 after N-cadherin pull-down ( Figure 1B, lane 4, arrowhead), but not with the control IgG serum ( Figure 1B, lane 3). These findings reveal N-cadherin and Cadherin-7 physically interact *in vivo.* As N-cadherin is noted in trigeminal placodal neurons and cranial mesenchyme ^16^ but only neural crest cells express Cadherin-7, ^14^ our data suggest heterophilic interactions between Cadherin-7 in neural crest cells and N-cadherin in placodal neurons and/or the mesenchyme.

### Cadherin-7 and N-cadherin form heterophilic interactions *in vitro*

**Figure 2.**
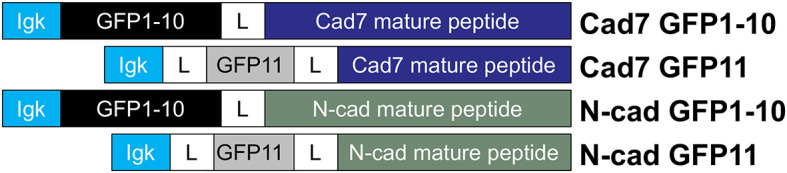
Cadherin-expressing GRASP constructs. Cartoon diagram showing GFP-cadherin fusion proteins that were constructed by joining the kappa light chain to distinct GFP subunits (1–10, or 11), followed by a linker region and then the mature cadherin peptide. GFP, green fluorescent protein; GRASP, GFP reconstitution across synaptic partners; N-cad, neural cadherin; Cad7, Cadherin-7.

Given the results of our pull-down experiments, we hypothesized that physical interactions between Cadherin-7 in neural crest cells and N-cadherin in placodal neurons mediate, in part, the successful aggregation of these cell types during trigeminal gangliogenesis. To address this, we adapted and modified a GRASP assay to evaluate interactions specifically between these two cadherins, both *in vitro* and *in vivo.* GRASP relies upon functional complementation ( *i.e.*, GFP fluorescence) between two non-fluorescing or split GFP fragments (GFP1-10, GFP11). Reconstitution of GFP can only occur when the split GFP molecules are in close proximity to each other, as observed in other systems that defined interactions between extracellular domains of two membrane proteins. ^23^^,^^30^^–^^32^ We designed Cadherin-7 and N-cadherin GRASP vectors ( Figure 2) with GFP subunits fused in frame to the respective cadherin extracellular domain (Cadherin-7 GFP1-10, Cadherin-7 GFP11, N-cadherin GFP1-10, N-cadherin GFP11; GenScript). Constructs were based on GRASP plasmids developed by the Sanes lab (Addgene), which generate intact GFP fluorescence due to neuroligin-neurexin interactions, with no GFP noted with single constructs. ^23^

**Figure 3.**
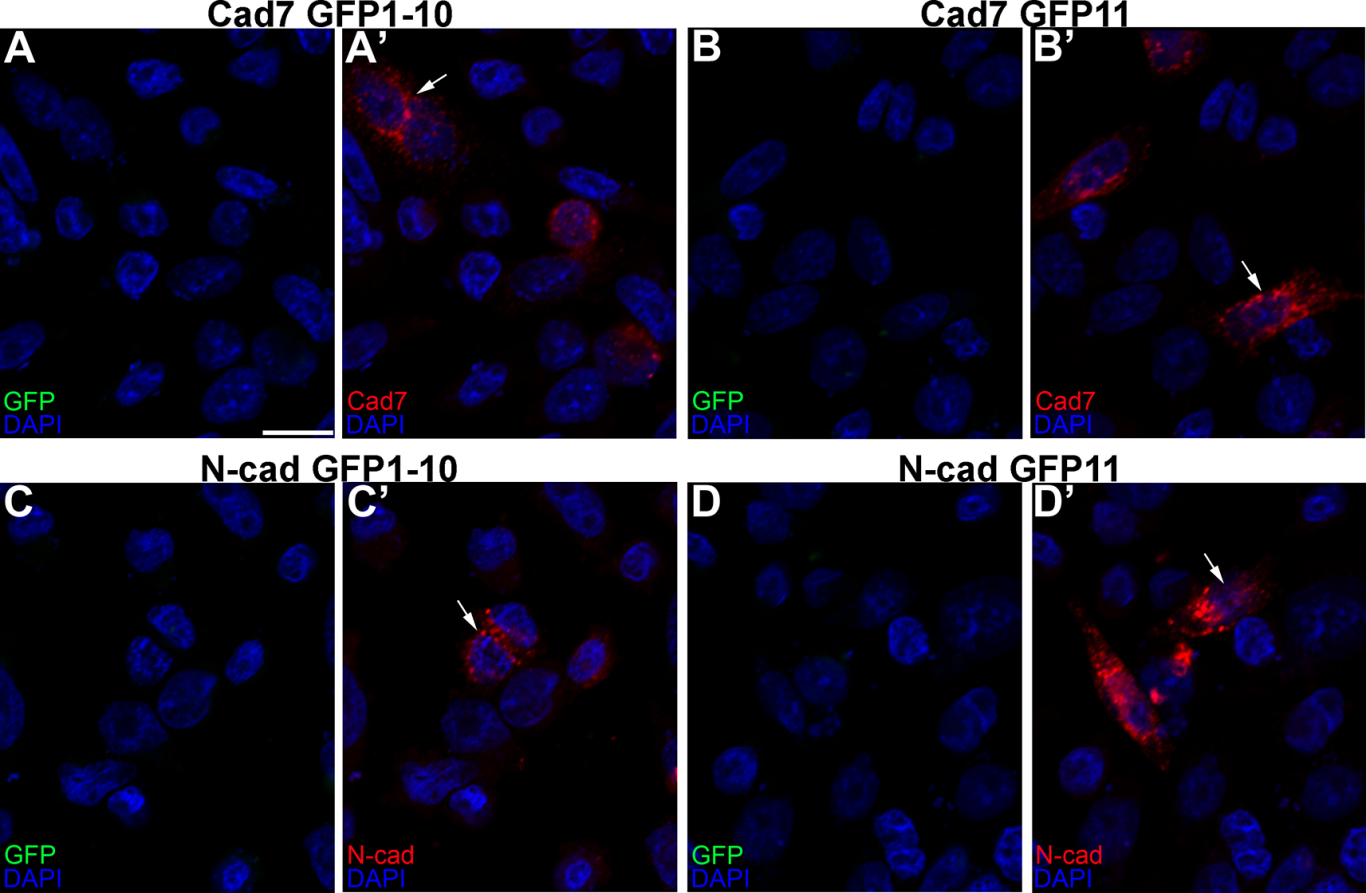
Individual cadherin GRASP constructs express their respective cadherins but not GFP. Single transfections of Cad7 GFP1-10 (A-A’), Cad7 GFP11 (B-B’), N-cad GFP1-10 (C-C’), and N-cad GFP11 (D-D’) were conducted in CHO cells, followed by immunocytochemistry for Cad7 (A’, B’, red) or N-cad (C’, D’, red). GFP fluorescence was also examined in the appropriate microscope channel (488) but not observed. Arrows point to cadherin expression in transfected cells. DAPI (blue), cell nuclei. N=3 replicates for all treatments. Scale bar in (A) is 50 μm and applies to all images. GFP, green fluorescent protein; GRASP, GFP reconstitution across synaptic partners; CHO, Chinese hamster ovary; N-cad, neural cadherin; Cad7, Cadherin-7.

**Figure 4.**
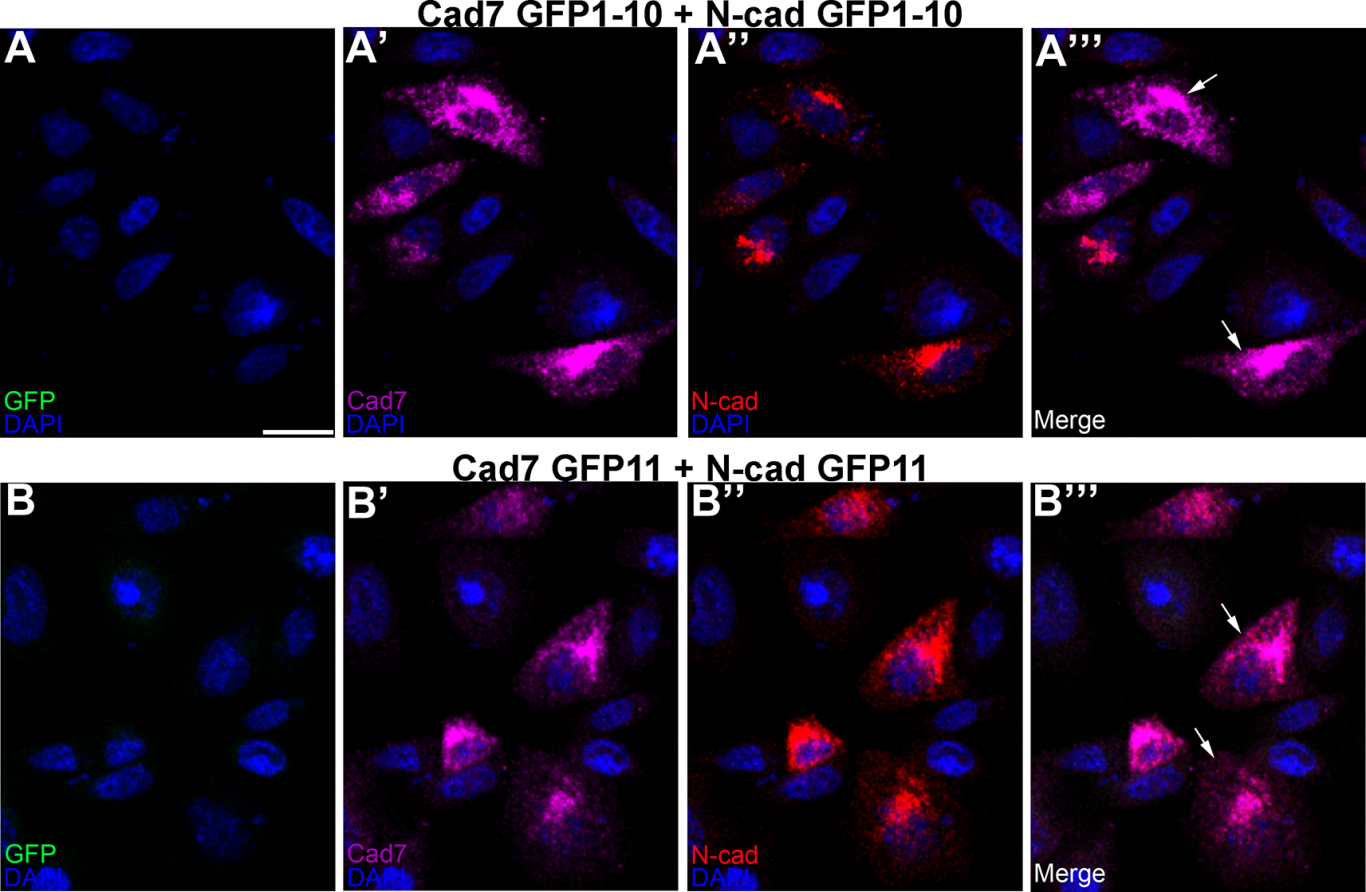
Different cadherin-expressing GRASP constructs possessing the same GFP domains do not reconstitute GFP. Co-transfection of CHO cells with Cad7 GFP1-10 + N-cad GFP1-10 (A-A”’), or Cad7 GFP11 + N-cad GFP11 (B-B”’), was performed, followed by immunocytochemistry for Cad7 (A’, A”’, B’, B”’, purple) and N-cad (A”, A”’, B”, B”’, red). GFP fluorescence was also examined in the appropriate microscope channel (488) but not observed. Arrows point to cadherin expression in co-transfected cells. DAPI (blue), cell nuclei. Scale bar in (A) is 50 μm and applies to all images. N=8 replicates for both treatments. GFP, green fluorescent protein; GRASP, GFP reconstitution across synaptic partners; CHO, Chinese hamster ovary; N-cad, neural cadherin; Cad7, Cadherin-7.

We first showed that all constructs expressed their respective cadherins by transfecting CHO cells, which lack endogenous cadherins, ^33^ with each GRASP construct, followed by immunostaining for each cadherin ( Figure 3A’, B’, C’, D’, arrows). Importantly, no GFP fluorescence was noted under any condition, as expected. We next evaluated the specificity of the split GFP moieties to generate GFP by co-transfecting CHO cells with the same split GFP constructs, but fused to a different cadherin ( *i.e.*, Cadherin-7 GFP1-10 and N-cadherin GFP1-10). In these control experiments, expression of each cadherin was observed once again ( Figure 4A”’, B”’, arrows), but no GFP was reconstituted, reinforcing the specificity of the assay.

**Figure 5.**
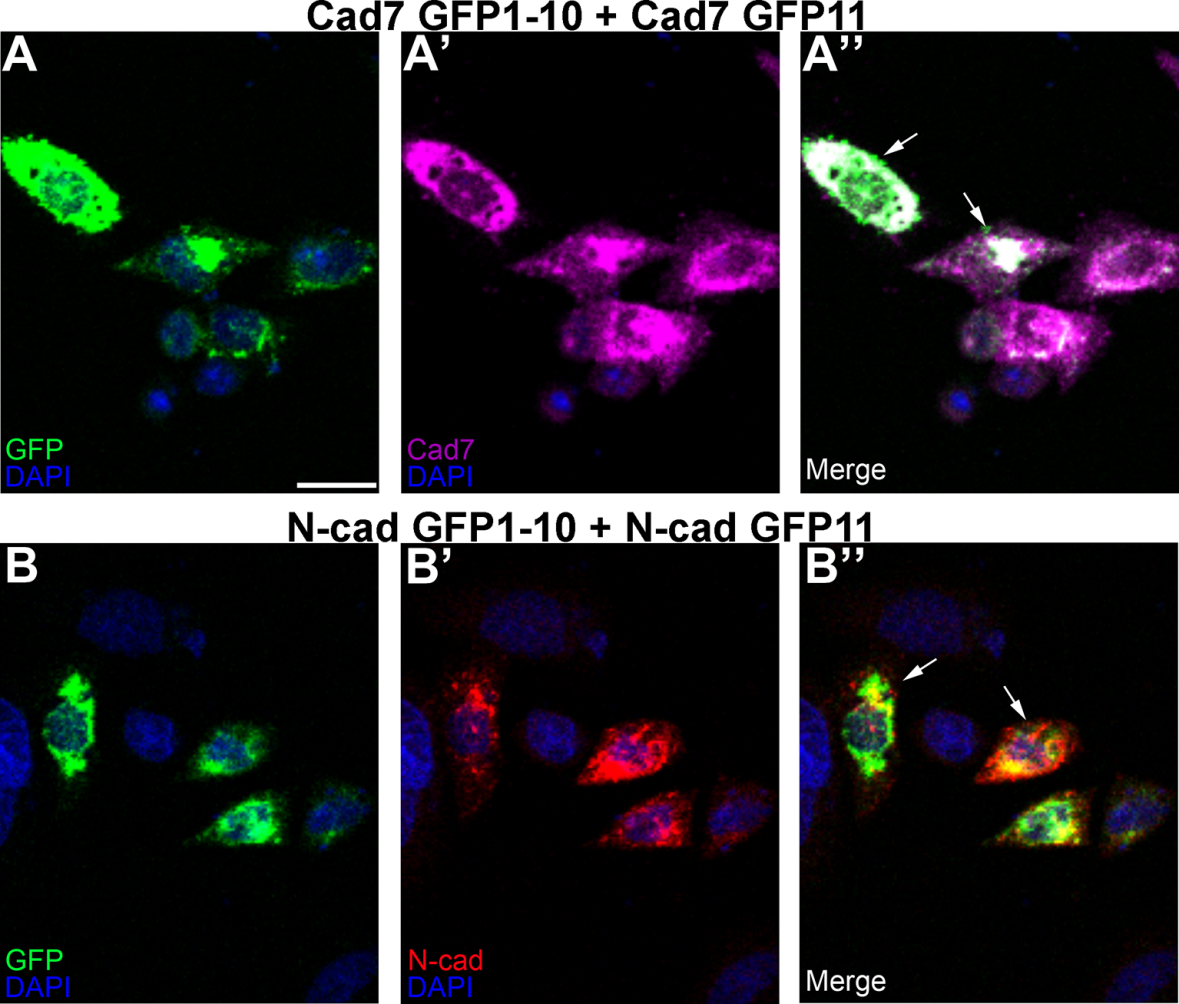
Identical cadherin-expressing GRASP constructs possessing complementary GFP domains reconstitute GFP in *cis.* CHO cells were co-transfected with complementary split GFP constructs expressing the same cadherin (Cad7 GFP1-10 and Cad7 GFP11 (A-A”); N-cad GFP1-10 and N-cad GFP11, (B-B”)), followed by immunocytochemistry for Cad7 (A’, A”, purple) or N-cad (B’, B”, red). GFP fluorescence was also examined in the appropriate microscope channel (488, A, A”, B, B”, green). Arrows point to GFP fluorescence in transfected cells, indicative of physical interactions between each split GFP-expressing cadherin. DAPI (blue), cell nuclei. Scale bar in (A) is 50 μm and applies to all images. N=8 replicates for both treatments. GFP, green fluorescent protein; GRASP, GFP reconstitution across synaptic partners; CHO, Chinese hamster ovary; N-cad, neural cadherin; Cad7, Cadherin-7.

Next, we addressed whether *cis* interactions between complementary split GFP constructs could generate an intact GFP molecule *in vitro.* To this end, we co-transfected CHO cells with complementary split GFP constructs expressing the same cadherin ( Figure 5) and examined GFP fluorescence and cadherin expression by immunostaining. GFP fluorescence was detected with both Cadherin-7- ( Figure 5A, A”, arrows) or N-cadherin- ( Figure 5B, B”, arrows) expressing split GFP constructs, along with expression of each respective cadherin ( Figure 5A’, B’), demonstrating effective GFP reconstitution *via* homophilic cadherin interactions. Further, these transfected cells exhibited a round morphology with few protrusions emanating from the cell surface, similar to that seen with control transfections.

**Figure 6.**
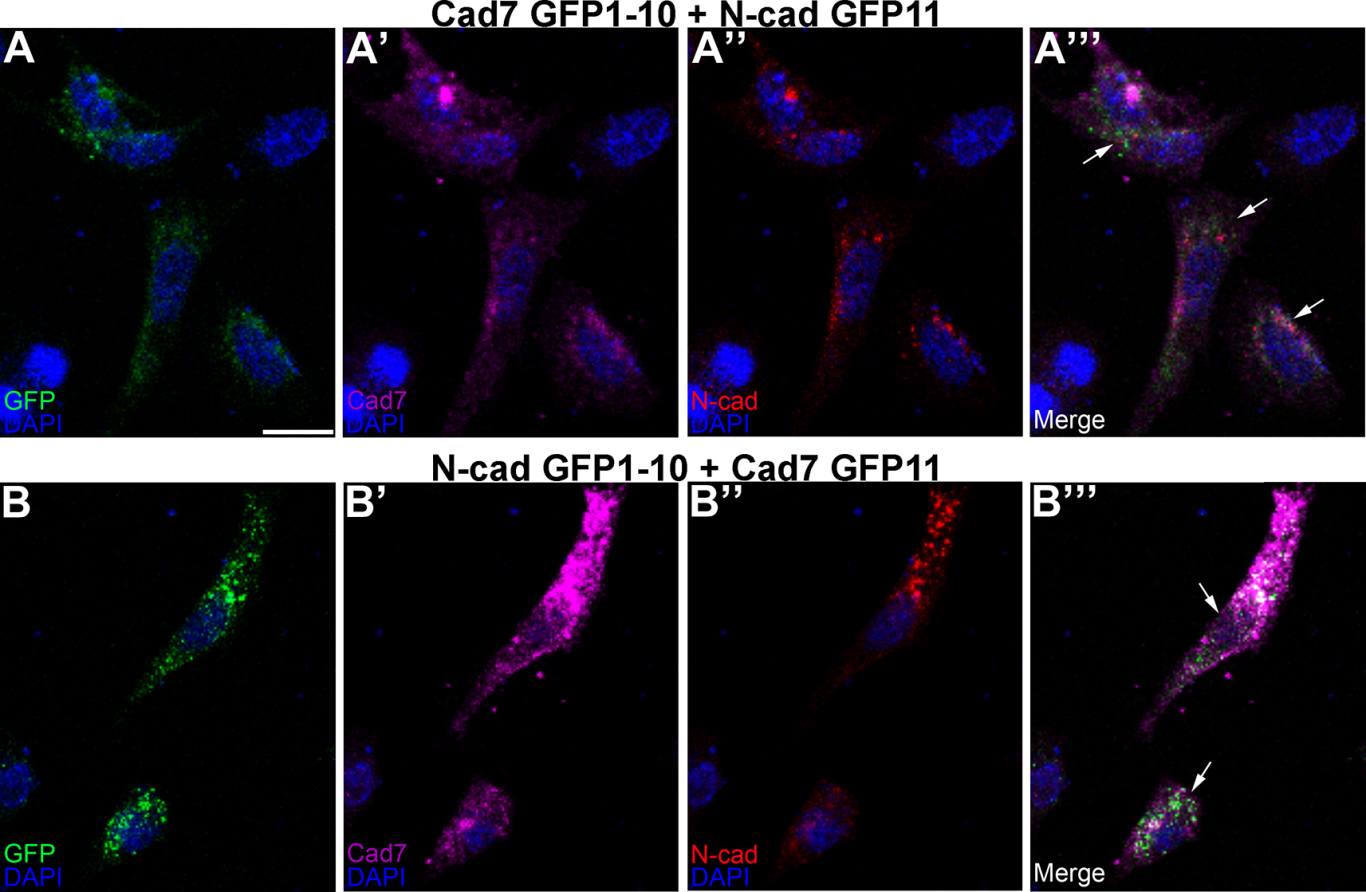
Different cadherin-expressing GRASP constructs possessing complementary GFP domains reconstitute GFP in *cis.* Cad7 GFP1-10 and N-cad GFP11 (A-A”’), or N-cad GFP1-10 and Cad7 GFP11 (B-B”’), were co-transfected into CHO cells, followed by immunocytochemistry for Cad7 (A’, A”’, B’, B”’, purple) and N-cad (A”, A”’, B”, B”’, red). GFP fluorescence was also examined in the appropriate microscope channel (488, A, A”’, B, B”’, green). Arrows point to GFP fluorescence in transfected cells, indicative of physical interactions between each split GFP-expressing cadherin. DAPI (blue), cell nuclei. Scale bar in (A) is 50 μm and applies to all images. N=8 replicates for both treatments. GFP, green fluorescent protein; GRASP, GFP reconstitution across synaptic partners; CHO, Chinese hamster ovary; N-cad, neural cadherin; Cad7, Cadherin-7.

To evaluate this in the context of the potential formation of heterophilic cadherin complexes, the same co-transfection experiment was conducted in CHO cells but this time using complementary split GFP constructs fused to a different cadherin ( Figure 6). Our results revealed GFP reconstitution ( Figure 6A, A”’, B, B”’, arrows) and cadherin expression ( Figure 6A- A”’, B- B”’), pointing to the ability of Cadherin-7 and N-cadherin to interact in *cis* and form heterophilic complexes, further validating our *in vivo* biochemistry results in the chick trigeminal ganglion. Interestingly, these transfected cells adopted a more fibroblastic, and often spindly, morphology, particularly when compared to cells transfected with homophilic cadherin split GFP-expressing constructs, which were rounder in appearance ( Figure 5).

Next, we calculated the percentage of GFP-positive cells in all of our transfection assays and observed a greater number of GFP-positive cells after transfection of homophilic split cadherin GFP constructs compared to that observed after transfection of heterophilic split cadherin GFP constructs ( Table 1). While there is no statistically significant difference in the percentage of GFP-positive cells in N-cad GFP1-10 + N-cad GFP11- versus Cad7 GFP1-10 + Cad711-transfected cells (p=0.19), we did find a statistically significant difference in the percentage of GFP-positive cells after transfection of heterophilic cadherin split GFP constructs. Transfection of Cad7 GFP1-10 + N-cad GFP11 gave rise to a 1.5-fold increase in the percentage of GFP-positive cells compared to N-cad GFP1-10 + Cad7 GFP11 (p=0.013).

**Table 1.** Transfection efficiency of Cadherin split GFP plasmids.

	Cad7 GFP1-10 + Cad7 GFP11	N-cad GFP1-10 + N-cad GFP11	Cad7 GFP1-10 + N-cad GFP11	Cad7 GFP11 + N-cad GFP1-10
Percent transfected	10.51±0.22% ^a^	13.22±0.91% ^a^	8.90±0.64% ^a^	5.76±0.88% ^b^

Note: Transfection efficiency was calculated as the number of GFP ^+^ cells/DAPI ^+^ cells±SEM. Differing superscripts denote statistically significant values (p<0.05).

### Physical interactions between neural crest cells and placodal neurons in the trigeminal ganglion are mediated, in part, by Cadherin-7 and N-cadherin

**Figure 7.**
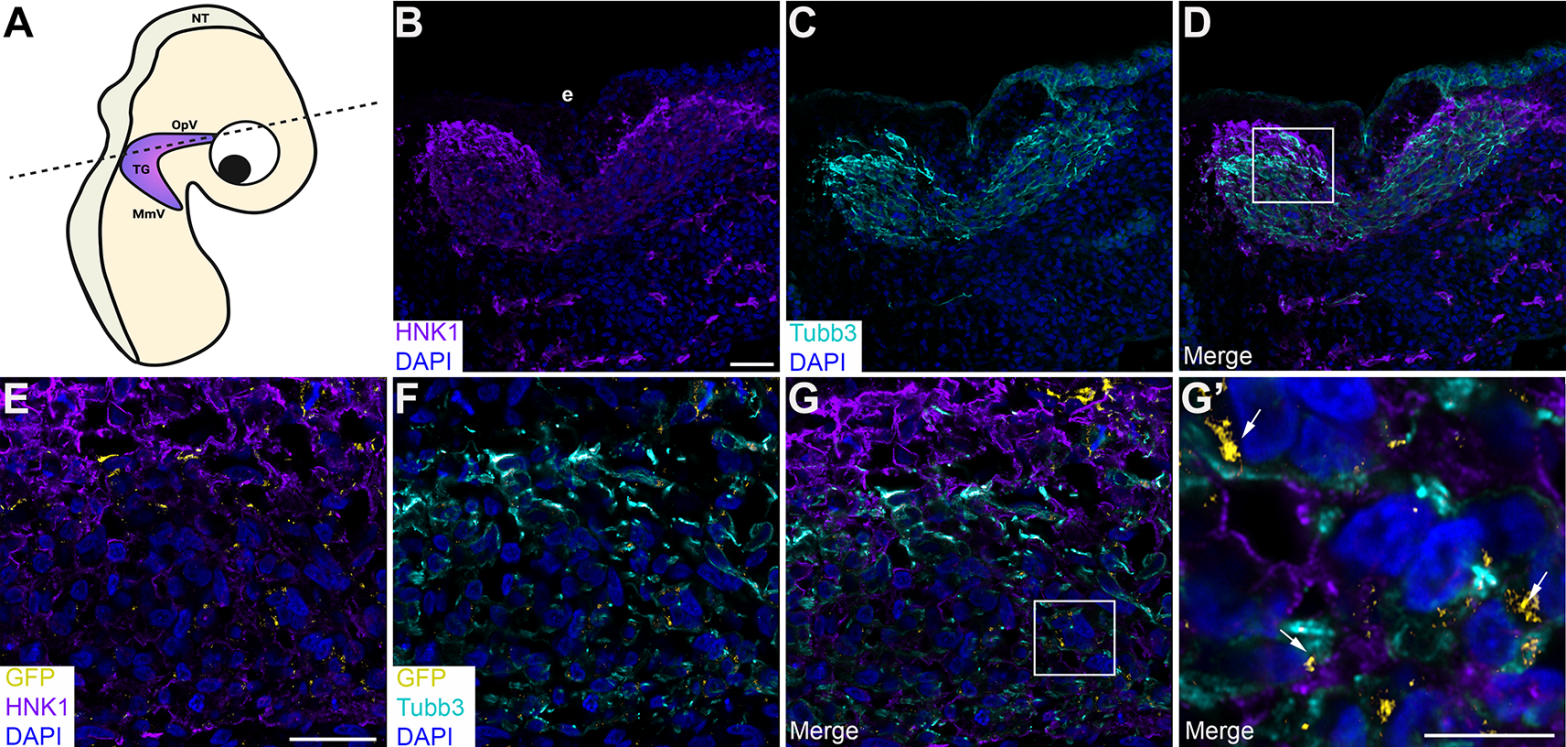
Cadherin-7 GFP1-10 and N-cadherin GFP11 form heterophilic interactions *in trans* in the trigeminal ganglion. (A) Cartoon diagram of a chick embryo showing the forming trigeminal ganglion, with the dotted line indicating the axial level at which images were captured. Diagram created with BioRender.com. Sequential electroporations in the chick embryo were conducted as follows: Premigratory neural crest cells were first electroporated with Cad7 GFP1-10 followed by electroporation of trigeminal placode cells with N-cad GFP11. Section immunohistochemistry for HNK1 (purple, marks neural crest cells; B, D, E, G, G’) and Tubb3 (cyan, marks neurons which are placode cell-derived at this stage; C, D, F, G, G’) was then performed. GFP signal (yellow, arrows, E-G’) was captured in the appropriate channel (488). (B-D) Representative sections that show the morphology of the trigeminal ganglion at this stage of development. (E-G) Higher magnification images of the boxed region in (D). (G’) Higher magnification image of the boxed region in (G), with GFP identified by arrows. DAPI (blue), cell nuclei. Scale bar in (B) is 100 μm and applies to (C-D); scale bar in (E) is 100 μm and applies to (F-G); and scale bar in (G’) is 50 μm. N=6 embryos. NT, neural tube; MmV, maxillomandibular lobe; OpV, ophthalmic lobe; TG, trigeminal ganglion; e, ectoderm; N-cad, neural cadherin; Cad7, Cadherin-7; GFP, green fluorescent protein; HNK-1, human natural killer-1; Tubb3, Tubulin beta-3 chain.

**Figure 8.**
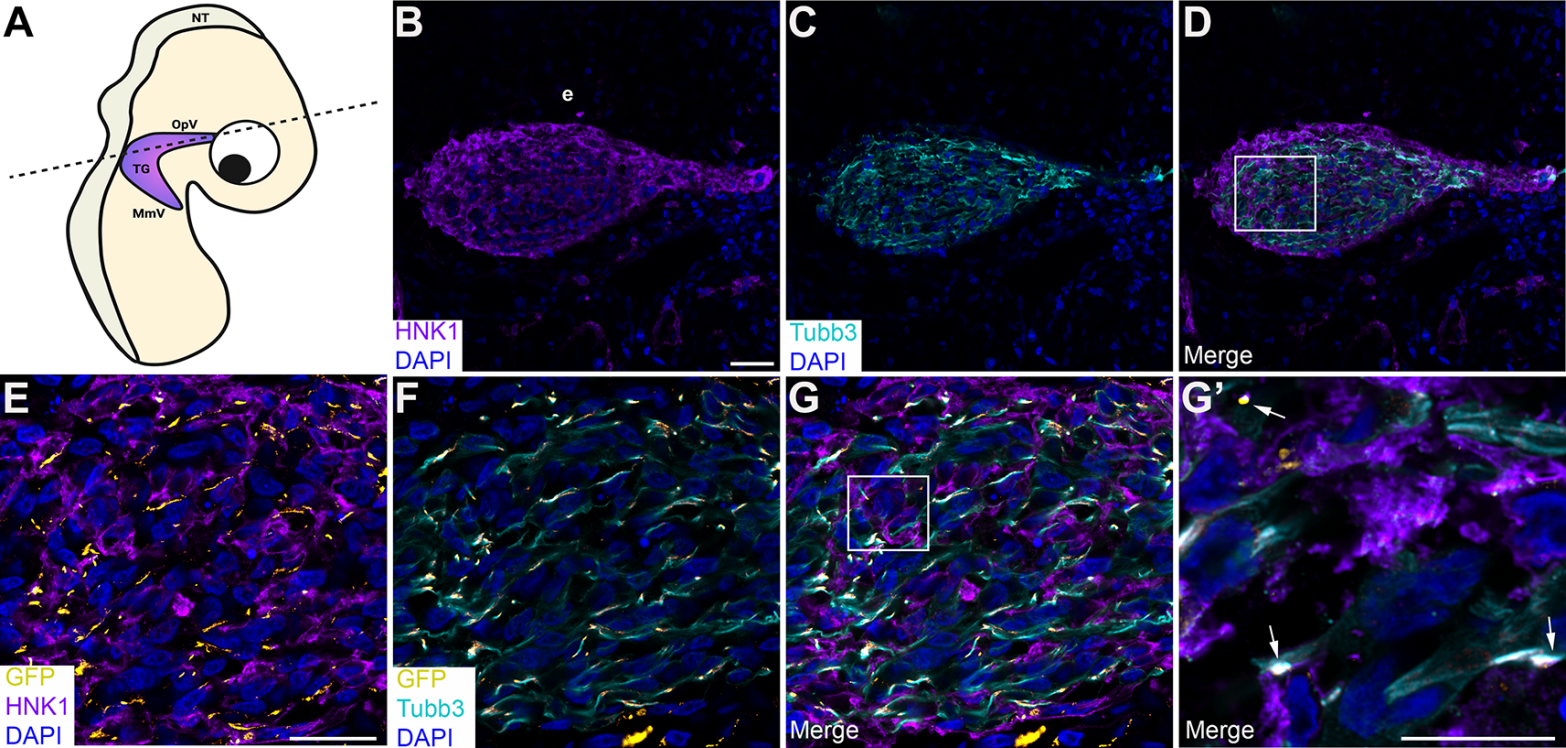
Cadherin-7 GFP11 and N-cadherin GFP1-10 form heterophilic interactions *in trans* in the trigeminal ganglion. (A) Cartoon diagram of a chick embryo showing the forming trigeminal ganglion, with the dotted line indicating the axial level at which images were captured. Diagram created with BioRender.com. Sequential electroporations in the chick embryo were conducted as follows: Premigratory neural crest cells were first electroporated with Cad7 GFP11 followed by electroporation of trigeminal placode cells with N-cad GFP1-10. Section immunohistochemistry for HNK1 (purple, marks neural crest cells; B, D, E, G, G’) and Tubb3 (cyan, marks neurons which are placode cell-derived at this stage; C, D, F, G, G’) was then performed. GFP signal (yellow, arrows, E-G’) was captured in the appropriate channel (488). (B-D) Representative sections that show the morphology of the trigeminal ganglion at this stage of development. (E-G) Higher magnification images of the boxed region in (D). (G’) Higher magnification image of the boxed region in (G), with GFP identified by arrows. DAPI (blue), cell nuclei. N=13 embryos. Scale bar in (B) is 100 μm and applies to (C-D); scale bar in (E) is 100 μm and applies to (F-G); and scale bar in (G’) is 50 μm. NT, neural tube; MmV, maxillomandibular lobe; OpV, ophthalmic lobe; TG, trigeminal ganglion; e, ectoderm; N-cad, neural cadherin; Cad7, Cadherin-7; GFP, green fluorescent protein; HNK-1, human natural killer-1; Tubb3, Tubulin beta-3 chain.

To corroborate our findings and examine cadherin intercellular interactions during trigeminal ganglion assembly *in vivo*, we turned to a sequential electroporation assay in which a Cadherin-7 split GFP construct was first electroporated into premigratory neural crest cells, followed by a second electroporation of a complementary N-cadherin split GFP construct to target trigeminal placode cells in the surface ectoderm ( Figures 7 and 8). Transverse sections taken from electroporated embryos were processed for immunohistochemistry to identify neural crest cells and placodal neurons within the forming trigeminal ganglion. Remarkably, we observed GFP-positive regions and/or puncta ( Figure 7E- G’; Figure 8E- G’; arrows) between neural crest cells (labeled by HNK-1; Figure 7B, D, E, G, G’; Figure 8B, D, E, G, G’) and placodal neurons (labeled by Tubb3; Figure 7C, D, F, G, G’; Figure 8C, D, F, G, G’) in the presence of the appropriate split GFP constructs. These data indicate Cadherin-7 and N-cadherin are in close proximity to interact in *trans* and permit the reconstitution of GFP *in vivo*, even in different cell types.

**Figure 9.**
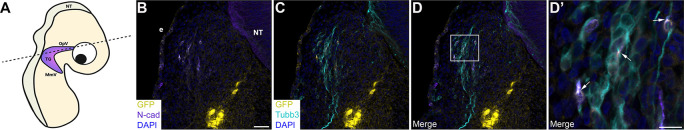
N-cadherin GRASP constructs expressing complementary GFP domains reconstitute GFP *in cis* in the trigeminal ganglion. (A) Cartoon diagram of a chick embryo showing the forming trigeminal ganglion, with the dotted line indicating the axial level at which images were captured. Diagram created with BioRender.com. To target trigeminal placode cells, ectodermal electroporations were conducted in the chick embryo with both N-cad GFP1-10 and N-cad GFP11 constructs. Section immunohistochemistry for N-cad (purple; B, D, D’) and Tubb3 (cyan, marks neurons placode cell-derived at this stage; C-D’) was then performed. GFP signal (yellow, arrows, B-D’) was captured in the appropriate channel (488). (B-D) Representative sections that show the morphology of the trigeminal ganglion at this stage in development. (D’) Higher magnification image of the boxed region in (D), with GFP and N-cad double-positive cells identified by arrows. Scale bar in (B) is 100 μm and applies to (C-D), and scale bar in (D’) is 50 μm. N=3 embryos. NT, neural tube; MmV, maxillomandibular lobe; OpV, ophthalmic lobe; TG, trigeminal ganglion; e, ectoderm; N-cad, neural cadherin; GFP, green fluorescent protein; Tubb3, Tubulin beta-3 chain.

Next, we quantified the number of GFP-positive regions/puncta in serial sections through the forming trigeminal ganglion in each sequential electroporation experimental condition. Remarkably, this analysis yielded similar results to what we observed *in vitro*, namely a 1.6-fold increase in the number of GFP-positive regions/puncta after sequential electroporation of Cad7 GFP1-10 + N-cad GFP11 compared to that seen in Cad7 GFP11 + N-cad GFP1-10-electroporated embryos (p=0.014, Table 2). In contrast, co-electroporation of N-cad GFP1-10 + N-cad GFP11 into trigeminal placode cells, followed by incubation of embryos to trigeminal ganglion-forming stages, yielded robust GFP fluorescence throughout the plasma membrane of placodal precursors ( Figure 9A- C) and neurons ( Figure 9D’, arrows). Quantification of the number of GFP-positive cells in these experiments revealed that 53% of the N-cadherin-positive trigeminal placode cells and neurons were also GFP-positive when split GFP construct electroporations were performed in *cis* ( Table 2). These data are consistent with those seen *in vitro* after transfection of the same cadherin split GFP construct combination. Together with our biochemistry data and results in cultured cells, our findings support the assertion that heterophilic interactions between Cadherin-7 in neural crest cells and N-cadherin in placodal neurons occur during trigeminal gangliogenesis.

**Table 2.** Electroporation efficiency of Cadherin split GFP plasmids.

	N-cad GFP 1-10; N-cad GFP 11	Cad7 GFP1-10 + N-cad GFP11	Cad7 GFP11 + N-cad GFP1-10
GFP-positivity	53±0.29%	16 regions±2.07 ^a^	10 regions±1.42 ^b^

Note: Column 1 denotes the co-electroporation efficiency of N-cad GFP1-10 and N-cad GFP11 plasmids into trigeminal placode cells, as calculated by the number of GFP ^+^ cells/N-cad ^+^ cells±SEM. Columns 2 and 3 report the average number of GFP-positive regions within a trigeminal ganglion section ± SEM. For the heterophilic cadherin/GRASP experiments, differing superscripts denote statistically significant values (p<0.05).

**Figure 10.**
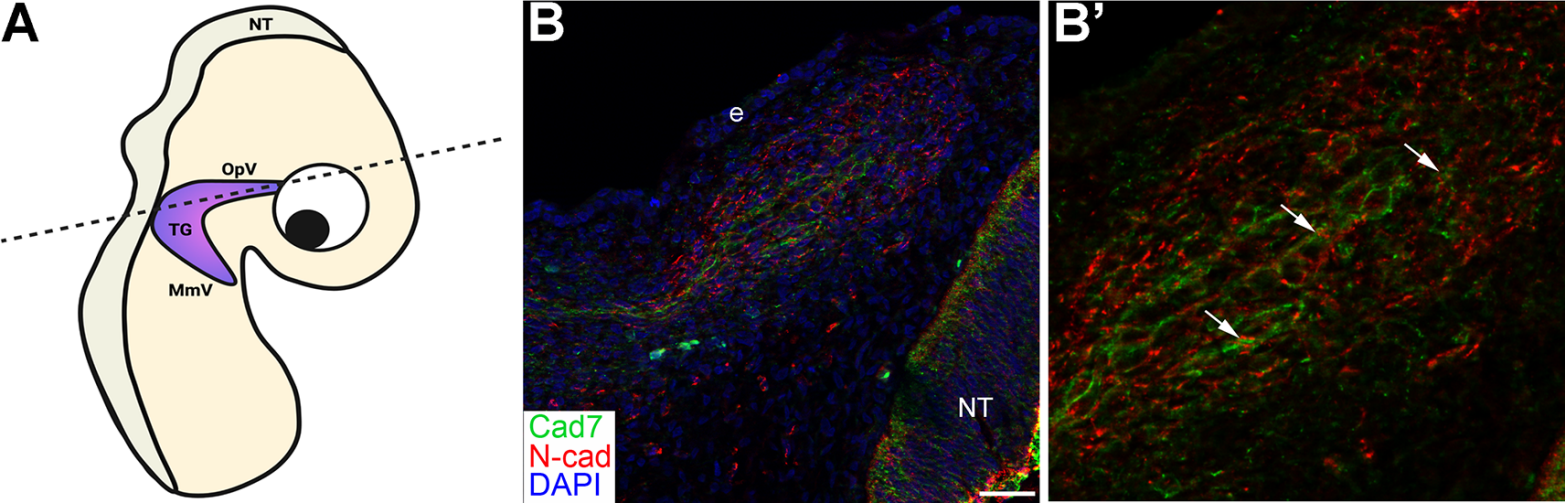
N-cadherin and Cadherin-7 are expressed in condensing placodal neurons and neural crest cells, respectively, during chick trigeminal ganglion assembly. (A) Cartoon diagram of forming TG in the developing chick embryo. Plane of section is shown by the dotted line. Diagram created with BioRender.com. (B) Representative transverse section taken through the forming TG followed by immunohistochemistry for Cad7 (green, marks neural crest cells) and N-cad (red, marks neurons which are all placode-derived at this stage). (C) Higher magnification (digital) of the TG in (B). DAPI (blue), cell nuclei. Scale bar in (B) is 100 μm and applies to (B’) but is 50 μm. N=5 embryos. TG, trigeminal ganglion; Cad7, Cadherin-7; N-cad, neural cadherin; NT, neural tube; OpV, ophthalmic branch; MmV, maxillomandibular branch; e, ectoderm.

Moreover, the preceding data are in keeping with the *in vivo* distribution of N-cadherin-expressing placodal neurons and Cadherin-7-expressing neural crest cells in the forming ganglion at this developmental stage ( Figure 10). Although it is evident that neural crest cells and placodal neurons are in close proximity and can interact ( Figure 10B’, arrows), the distribution of these cells also reveals that homophilic cadherin interactions are likely to occur within each cell type proper (i.e., neural crest cell-neural crest cell, placodal neuron-placodal neuron) as the ganglion begins to assemble. Collectively, our *in vivo* results support a role for neural crest cell-placode cell interactions during early trigeminal ganglion development.

## Discussion

Cranial neural crest cells and placode cells initially form in close proximity but become spatially separated as development ensues. ^8^^,^^34^^–^^36^ While these cells give rise to distinct derivatives, they will both form sensory neurons of the trigeminal ganglion, innervating much of the head and face to relay information related to pain, touch, and temperature to the central nervous system. ^3^^–^^5^ The cellular origin of the trigeminal ganglion has been known for decades ^7^^,^^10^^,^^37^; however, molecular mechanisms mediating early interactions between neural crest cells and placodal neurons to build the trigeminal ganglion have not been well characterized. In the chick embryo, studies uncovered the importance of cadherin-mediated interactions, as distinct cadherins are expressed by neural crest cells (Cadherin-7) ^13^^,^^14^ and placode cells and their neuronal derivatives (N-cadherin) during early trigeminal ganglion assembly. ^16^ The presence of two different cadherins on these coalescing cells begs the question as to whether heterophilic interactions exist between them to allow for proper trigeminal ganglion formation, particularly since cells expressing these cadherins can form mixed aggregates *in vitro*. ^13^

Our studies now address this question through the use of biochemistry and an adapted GRASP assay to examine cadherin interactions during trigeminal ganglion development. Through *in vitro* transfection experiments and use of embryonic trigeminal ganglia tissue, we demonstrate a physical interaction between Cadherin-7 and N-cadherin. This is the first report to reveal, biochemically, that Cadherin-7-N-cadherin complexes can form and, notably, are present while the trigeminal ganglion assembles. While we cannot rule out the presence of other protein(s) in the embryo to serve as a “bridge” to allow these cadherins to associate, these data still provide strong evidence that these interactions do exist *in vivo.*

To generate the trigeminal ganglion, Cadherin-7-expressing cranial neural crest cells first migrate through the embryonic mesenchyme to the trigeminal ganglionic anlage. Here, they intermingle with newly differentiated, N-cadherin-expressing trigeminal placode-derived neurons, which have delaminated from the surface ectoderm and have also migrated through the mesenchyme. Since the cranial mesenchyme expresses N-cadherin, it is possible that the Cadherin-7-N-cadherin complexes we detected through our biochemistry studies represent interactions between Cadherin-7 on neural crest cells and N-cadherin expressed in mesenchymal cells. However, based upon the abundance of neurons in relation to the mesenchyme in dissected trigeminal ganglia, we think the primary source of N-cadherin in these interactions comes from the placodal neurons. Moreover, migratory neural crest cells form “corridors” through which placodal neurons migrate, as they provide a more permissive substrate for migration than the surrounding cranial mesenchyme. ^38^^,^^39^ As such, neural crest cells and placodal neurons are tightly juxtaposed during the assembly of the trigeminal ganglion, making it more likely that the interactions we are detecting arise from Cadherin-7 on neural crest cells and N-cadherin on placodal neurons.

To further define and directly visualize these heterophilic cadherin interactions, we conducted a GRASP assay in cell culture and in the embryo. We generated two split GFP constructs (GFP domains 1-10 or GFP domain 11) fused to both Cadherin-7 and N-cadherin and examined the ability of these cadherins to associate in *cis* and in *trans* to generate GFP. Through cell culture co-transfection experiments, we demonstrated that GFP could be reconstituted as long as the split GFP constructs were complementary, providing further evidence that Cadherin-7 and N-cadherin can interact in *cis.* Importantly, no GFP was generated after co-transfection of like split GFP moieties fused to different cadherins, pointing to the specificity of the GFP reconstitution.

Intriguingly, we noted that transfection of cells with like cadherins and complementary GRASP constructs gave rise to cells that often exhibit a round shape or are only somewhat fibroblastic, with few protrusions emanating from the cell ( Figure 5). Conversely, transfection of cells with different cadherins and complementary GRASP constructs caused cells to adopt a much more fibroblastic, and often spindly, morphology. This could be due to the presence of both a Type I (N-cadherin) and Type II (Cadherin-7) cadherin in these cells. A parallel to this can be found *in vivo* with respect to the overlapping expression domains of *N-cadherin* and *Cadherin-7* in the developing chick spinal cord. ^40^ These neuroepithelial cells are organized in a pseudostratified manner and thus exhibit a spindly morphology as they are densely packed within the neural tube/forming spinal cord.

Quantification of GFP in our transfection experiments led to the conclusion that more GFP-positive cells were present upon transfection of complementary split GFP constructs fused to the same cadherin (homophilic interactions) compared to transfection of complementary split GFP constructs fused to different cadherins (heterophilic interactions). These findings are consistent with measurements of dissociation constants in homophilic and heterophilic cadherin cell aggregates *in vitro*, which revealed that homophilic cadherin interactions are stronger than heterophilic interactions. ^41^ Moreover, we find that the number of GFP-positive cells is highest in cells transfected with split GFP constructs fused to N-cadherin compared to Cadherin-7. These results are in keeping with a report showing that N-cadherin confers a much higher degree of adhesivity compared to Cadherin-7. ^42^

Finally, we noted a statistically significant 1.5-fold increase in GFP-positive cells upon transfection with Cad7 GFP1-10 + N-cad GFP11 compared to Cad7 GFP11 + N-cad GFP1-10 (p=0.013). This trend is also observed *in vivo* (see below). While we might expect these numbers in our heterophilic experiments to be similar, the difference could be explained by the inability of cells to effectively express N-cad GFP1-10, or, conversely, the capacity of cells to more readily express Cad7 GFP11. We favor the former idea, however, for the following reasons. Given that the *N-cadherin* coding sequence is larger than the *Cadherin-7* coding sequence (~300 nucleotides difference), coupled with the greater size of the split GFP1-10 moiety, it is possible that cells do not readily express N-cad GFP1-10 (compared to N-cad GFP11). Further, since homophilic cadherin interactions are stronger and more stable than heterophilic ones, ^41^ heterophilic interactions are, by nature, more transient. As such, the GFP we observe is a direct readout of these cadherin interactions and is correlated with their strength. Thus, we would expect homophilic interactions to yield the most GFP signal, with heterophilic interactions following next, as noted in both our *in vitro* and *in vivo* experiments. If cells do have more difficulty expressing N-cad GFP1-10, we would expect the GFP signal to be stronger in cells expressing Cad7 GFP1-10 + N-cad GFP11 versus those expressing Cad7 GFP11 + N-cad GFP1-10, which is in keeping with our results. 

With these tools, we next explored the ability of Cadherin-7-expressing neural crest cells to associate with N-cadherin-expressing placodal neurons. Sequential electroporation experiments were conducted in which complementary split GFP constructs were introduced into neural crest cells (Cadherin-7 split GFP construct) followed by placode cells (N-cadherin split GFP construct). Because of the anatomy of the chick embryo at the time of electroporation and tissue of origin of neural crest cells (dorsal neural folds) and placode cells (surface ectoderm), we can precisely, and independently, target each cell type. Notably, we observed GFP fluorescence at sites where neural crest cells and placodal neurons come into contact, visualized on sections taken through the developing trigeminal ganglion. These data reveal that Cadherin-7 and N-cadherin can interact in *trans* in different cell populations, providing insight into the ability of different cadherin-expressing cells to associate *in vivo.*

Although the number of GFP-positive regions was not extraordinarily high in this *in vivo* assay, this is to be expected given the nature of the electroporation, in which only a small amount of each split GFP construct was electroporated into each cell type in order to avoid potential artifacts of overexpression. Quantification of the number of GFP-positive regions/puncta through serial trigeminal ganglion sections, however, revealed results that were in keeping with what we observed *in vitro*. Specifically, we noted a statistically significant 1.6-fold increase in the GFP fluorescence reported in Cad7 GFP1-10 + N-cad GFP11-electroporated embryos vs. Cad7 GFP11 + N-cad GFP1-10-electroporated embryos (p=0.014). These results align with what we observe *in vitro*, with these differences also possibly arising for similar reasons as outlined above (e.g., ability of cells to effectively express N-cad GFP1-10). Alternatively, there might be inherent differences in the ability of neural crest cells and placode cells to transcribe and translate expression constructs like these, with neural crest cells more easily expressing Cad7 GFP1-10 compared to placode cells expressing N-cad GFP1-10. Finally, the heterophilic interactions we are examining occur in *trans*, not in *cis* as in our *in vitro* experiments. As such, they are dependent upon a cadherin split GFP construct getting not only electroporated but also appropriately trafficked, and correctly targeted, to a region of the plasma membrane, where it will then be in close proximity to a complementary cadherin split GFP construct on the other cell type. For these reasons, fewer heterophilic interactions may ensue in a given electroporated tissue. On the other hand, electroporation of N-cad GFP1-10 + N-cad GFP11 into placode cells results in robust GFP fluorescence, as these electroporations are performed in *cis*.

Other pathways have been discovered to regulate cellular interactions occurring during initial chick trigeminal ganglion coalescence, including Slit1-Robo2, ^11^^,^^16^ Wnt, ^43^^,^^44^ Neuropilin/Semaphorin, ^45^^,^^46^ and various growth factors ^47^ ( *e.g.*, Platelet-Derived Growth Factor ^48^), with many of these also identified in the developing mouse trigeminal ganglion. ^49^^,^^50^ In chick embryos, Robo2 signaling likely modulates levels of N-cadherin post-translationally, but the mechanisms underlying this are still not well characterized. Upstream pathways regulating Cadherin-7 expression in neural crest cells also remain obscure, but it is plausible that the preceding signal transduction pathways could impact the expression of Cadherin-7 and/or N-cadherin during trigeminal gangliogenesis. Future studies aimed at addressing this question will provide important insights into the regulation of neural crest-placodal neuron migration and adhesion.

The juxtaposition of Cadherin-7-expressing neural crest cells and N-cadherin-expressing placodal neurons in the forming trigeminal ganglion hinted at the possibility that heterophilic interactions between these two cadherins could, in part, mediate this process. While the functional roles of each cadherin in trigeminal ganglion assembly have been well described, less attention was paid to the importance of their expression in building the ganglion. Cultured cells expressing either Cadherin-7 or N-cadherin can form intermingled aggregates, ^13^ supporting the notion of heterophilic interactions and further assessed herein, but it was not evaluated *in vivo* until our studies. We now provide data uncovering a physical interaction between Cadherin-7 in neural crest cells and N-cadherin in placodal neurons within the trigeminal ganglion. Altogether, these findings shed light on the molecular mechanisms underscoring intercellular interactions requisite for trigeminal ganglion assembly during early chick embryonic development.

## Data availability

### Underlying data

Digital Repository at the University of Maryland, Animal & Avian Sciences Research Works: Neural crest cell-placodal neuron interactions are mediated by Cadherin-7 and N-cadherin during early chick trigeminal ganglion assembly. https://doi.org/10.13016/llyh-dppy. ^51^

This project contains the following underlying data:

• Figure 1: Raw western blot data (Original raw tiff files for the immunoblotting experiments)
• Figure 2: Plasmids.pdf (GRASP cadherin plasmid sequences)
• Figure 3: Transfection images for single split GFP cadherin constructs
• Figure 4: Transfection images for double, non-complementary, split GFP constructs with different cadherins
• Figure 5: Transfection images for double, complementary, split GFP constructs with the same cadherin
• Figure 6: Transfection images for double, complementary, split GFP constructs with different cadherins
• Figure 7: Tissue section images following electroporation of complementary split GFP constructs into neural crest cells and placode cells
• Figure 8: Tissue section images following electroporation of complementary split GFP constructs into neural crest cells and placode cells
• Figure 9: Tissue section images following electroporation of complementary split GFP constructs into placode cells
• Figure 10: Tissue section images following immunohistochemistry to label neural crest cells and placode cells
• Table 1: Excel file showing quantification of GFP fluorescence after co-transfection of complementary, split GFP constructs with the same or different cadherin *in vitro*
• Table 2: Excel file showing quantification of GFP fluorescence after co- or sequential electroporation of complementary, split GFP constructs with the same or different cadherin *in vivo*

Data are available under the terms of the Creative Commons Attribution 4.0 International license (CC-BY 4.0).
