# Supplementary material for: Neural crest cell-placodal neuron interactions are mediated by Cadherin-7 and N-cadherin during early chick trigeminal ganglion assembly
Source: F1000Res. 2022 Dec 12;11:741. Originally published 2022 Jul 4. [Version 2] doi: 10.12688/f1000research.122686.2 (PMC9475207; doi:10.12688/f1000research.122686.2)
Supplement: Peer review history

# Peer review: Reviewer response for version 2

**DOI:** 10.5256/f1000research.141659.r158007

**Published:** 2022-12-19

**Authors:** Paolo E. Forni, Ed Zandro Taroc

**Affiliations:** 1 Department of Biological Sciences, University at Albany, Albany, NY, USA; 2 The RNA Institute, University at Albany, Albany, USA

The authors addressed my concerns; I have no further comments to make.

Is the work clearly and accurately presented and does it cite the current literature?

Yes

If applicable, is the statistical analysis and its interpretation appropriate?

Partly

Are all the source data underlying the results available to ensure full reproducibility?

Yes

Is the study design appropriate and is the work technically sound?

Yes

Are the conclusions drawn adequately supported by the results?

Yes

Are sufficient details of methods and analysis provided to allow replication by others?

Yes

Reviewer Expertise:

Developmental neurobiology

We confirm that we have read this submission and believe that we have an appropriate level of expertise to confirm that it is of an acceptable scientific standard.

# Peer review: Reviewer response for version 2

**DOI:** 10.5256/f1000research.141659.r158006

**Published:** 2022-12-13

**Authors:** Susan Wray

**Affiliations:** 1 Cellular and Developmental Neurobiology Section, National Institute of Neurological Disorders and Stroke, National Institutes of Health, Bethesda, MD, USA

The authors have addressed all of my comments and the resulting revised manuscript is nicely improved.

Is the work clearly and accurately presented and does it cite the current literature?

Yes

If applicable, is the statistical analysis and its interpretation appropriate?

Not applicable

Are all the source data underlying the results available to ensure full reproducibility?

Yes

Is the study design appropriate and is the work technically sound?

Partly

Are the conclusions drawn adequately supported by the results?

Yes

Are sufficient details of methods and analysis provided to allow replication by others?

Yes

Reviewer Expertise:

GnRH neuroendocrine cells and  olfactory placode development

I confirm that I have read this submission and believe that I have an appropriate level of expertise to confirm that it is of an acceptable scientific standard.

# Peer review: Reviewer response for version 1

**DOI:** 10.5256/f1000research.134708.r148069

**Published:** 2022-09-14

**Authors:** Susan Wray

**Affiliations:** 1 Cellular and Developmental Neurobiology Section, National Institute of Neurological Disorders and Stroke, National Institutes of Health, Bethesda, MD, USA

This paper examines interactions between Cad-7 (expressed in neural crest cells) and N-cadherin (expressed in placodal cells) during formation of the trigeminal ganglia. Previous work from this lab showed that depletion of Cad-7 in neural crest cells alters placodal cell shape/orientation while overexpression changed trigeminal structure. The current study examines whether heterotypic interactions between these two cadherins occur. Using IP biochemical methods on trigeminal ganglion tissue, the authors show that Cad-7 can interact with N-cadherin. In addition, experiments using ‘split GFP’ fragments in the developing chick trigeminal ganglion are provided that support the IP results. Together the data strengthen the argument that heterotypic interactions are involved in trigeminal gangliogenesis.

 Clarifications/revisions that would strengthen the manuscript.

1.  Figure 6. It appears that the N-cad GFP1-10 + Cad7 GFP11 had more robust GFP puncta than the reverse group. Was this noticed in the trigeminal experiments? This brings up the issue of GFP quantification. Some measurements in both the cell lines as well as *in vivo* would be informative.
2.  Expression of GFP in ganglia electroporated with GRASP constructs. This is a very interesting experiment. However, it is difficult to understand all that is going on in Fig. 7. First, the methods state that the confocal imaging was performed at 20X magnification. Thus, I believe the images in Figure 7A”’ and B’’’ are enlargements of the images in A” and B”. To resolve where the GFP puncta occur, 60X or 100X may be required, as well as a few non-stacked single Z-plans. This would allow the reader to see where the combination is occurring. Second, the number of GFP expressing puncta is very low. This is dealt with in the discussion by saying “ *Although the number of GFP puncta was not extraordinarily high, this is to be expected given the nature of the electroporation, in which only a small amount of each split GFP construct was electroporated into each cell type in order to avoid potential artifacts of overexpression.” *However, a control experiment to aid the reader would be an additional group that receives PCN-cad GFP1-10 + PCN-cad GFP11 at the same ‘concentration’ as electroporated for the two mixed GRASPs. Minor changes to improve this figure would be to place all labels on white or black inset, since difficult to see staining labels in most panels and in A and B”’ to include DAPI to show cell bodies which would aid the reader to understand where GFP is being detected.

Below are some minor issues/suggestions.

 Introduction –

- The trigeminal ganglion, the largest of the cranial ganglia, contains three sensory branches…. is the clause necessary for the reader to know? Could be deleted making read through easier.
- revision suggested - More recent studies of confirmed Cadherin-7 protein in chick migratory cranial neural crest cells contributing to the trigeminal ganglion. ^14^ *These findings further clarify the reciprocal relationship observed*

Ethical Approval - I believe only these two sentences are needed

- No ethical approval was required for this study for the chick embryos. The NIH Office for Protection from Research Risks has interpreted “live vertebrate animal” to apply to avians ( *e.g.*, chick embryos) only after hatching.” Since our work does not utilize hatched chicks, no Institutional Animal Care and Use protocol for this work is necessary.

Is the work clearly and accurately presented and does it cite the current literature?

Yes

If applicable, is the statistical analysis and its interpretation appropriate?

Not applicable

Are all the source data underlying the results available to ensure full reproducibility?

Yes

Is the study design appropriate and is the work technically sound?

Partly

Are the conclusions drawn adequately supported by the results?

Yes

Are sufficient details of methods and analysis provided to allow replication by others?

Yes

Reviewer Expertise:

GnRH neuroendocrine cells and  olfactory placode development

I confirm that I have read this submission and believe that I have an appropriate level of expertise to confirm that it is of an acceptable scientific standard, however I have significant reservations, as outlined above.

# Response

**Published:** 2022-11-29

**Authors:** Lisa Taneyhill

**Affiliations:** University of Maryland, College Park, USA

We thank Dr. Wray for their insightful comments on the manuscript. We have responded to each point below and have made necessary updates to the manuscript, where applicable.

1. All Reviewers raised issues with the lack of quantification and statistics in the manuscript. To address this, we have now quantified GFP-positive puncta in our *in vivo* experiments. This was made possible by changing antigen colors in the images (as per Dr. Forni’s suggestion) and using a software program (Squassh plugin, FIJI) to quantify GFP-positive cells or regions between cells. Our data reveal a statistically significant increase in the number of GFP-positive regions in the Cad7 GFP1-10 + N-cad GFP11 electroporated embryos vs. the Cad7 GFP11 + N-cad GFP1-10 electroporated embryos (p = 0.014). We discuss possible reasons for this in the Discussion section, speculating that the reduced number of GFP-positive regions in the latter could be due to the general larger size of the N-cad GFP1-10 plasmid, potentially precluding robust expression of this construct, and/or intrinsic differences in the ability of neural crest and placode cells to transcribe and translate expression constructs. Although it was not possible to distinguish individual GFP-positive puncta/regions in the cell culture experiments, we have reported the percentage of GFP-positive cells in this assay, along with statistical comparisons across treatments. While there is no statistically significant difference in the number of GFP-positive cells after transfection of homophilic split GFP constructs (i.e., Cad7 GFP1-10 + Cad7 GFP11 or N-cad GFP1-10 + N-cad GFP11), we note a statistically significant increase in the number of GFP-positive cells in Cad7 GFP1-10 + N-cad GFP11-transfected cells vs. Cad7 GFP11 + N-cad GFP1-10-transfected cells (p = 0.013). These findings are in keeping with our *in vivo* results and have all been incorporated into the revised manuscript as part of new Table 1.
2. We appreciate the suggestion from Dr. Wray to compare the results from our sequential electroporation experiments of cadherin split GFP construct to those obtained after co-electroporation of N-cad GFP1-10 + N-cad GFP11 into placode cells. We have performed this experiment and quantified the number of GFP-positive cells, noting that such homophilic, *cis* interactions are more robust than those observed in our sequential electroporation experiments. These data are now provided as new Figure 9 and Table 2.
3. As per the suggestion of both Dr. Forni and Dr. Wray, we have placed the antigen names for new Figures 7-9 on a solid background. In addition, we have included the DAPI staining for the *in vivo* GRASP experiments to better visualize cell bodies and GFP detection.
4. We thank Dr. Wray for providing suggestions for text modifications for the Introduction and Ethical Approval sections and have made these changes in the revised manuscript.

# Peer review: Reviewer response for version 1

**DOI:** 10.5256/f1000research.134708.r147854

**Published:** 2022-09-13

**Authors:** Paolo E. Forni, Ed Zandro Taroc

**Affiliations:** 1 Department of Biological Sciences, University at Albany, Albany, NY, USA; 2 The RNA Institute, University at Albany, Albany, USA

This research report by Caroline A. Halmi and coworkers explored the potential formation of Cadherin-7-N-cadherin complexes during trigeminal ganglion assembly. The authors suggested that the neural crest-derived cells bind to the placodal-derived neurons via heterophilic Cadherin-7-N-cadherin binding. To test this hypothesis the authors adopted classic pull-down experiments and an elegant GRASP assays approach. The latter was used both in cell culture and in the embryos. The GRASP assay was designed in order to express complementary split GFP constructs into neural crest cells (Cadherin-7 split GFP construct) and placode cells (N-cadherin split GFP construct). In this type of assay, GFP can only be detected when the split fusion proteins directly interact. The data presented are compelling and the conclusions of the paper are relevant and of broad interest to the community.

 I suggest some changes that would improve the quality of the manuscript:

- All the figures would benefit from some quantifications and statistics supporting the data shown in the images. Moreover, in the legends, the authors should indicate the number of replicates.
- Fig.7 The quality of the pictures is not impressive. The combination of colors Red/magenta/green in A’’ A’’’ and B’’ B’’’ is very hard to read. The author should try different color combinations. The antigen names on the figures are hard to see, consider using a bold font and outline. Moreover, it would be good to have a low-mag image and diagram illustrating where the image was taken in context to the developing chick head. This would help the readers to understand the morphology of the region being described.

Is the work clearly and accurately presented and does it cite the current literature?

Yes

If applicable, is the statistical analysis and its interpretation appropriate?

Partly

Are all the source data underlying the results available to ensure full reproducibility?

Yes

Is the study design appropriate and is the work technically sound?

Yes

Are the conclusions drawn adequately supported by the results?

Yes

Are sufficient details of methods and analysis provided to allow replication by others?

Yes

Reviewer Expertise:

Developmental neurobiology

We confirm that we have read this submission and believe that we have an appropriate level of expertise to confirm that it is of an acceptable scientific standard, however we have significant reservations, as outlined above.

# Response

**Published:** 2022-11-29

**Authors:** Lisa Taneyhill

**Affiliations:** University of Maryland, College Park, USA

We thank Dr. Forni for their insightful comments on the manuscript. We have responded to each point below and will make necessary updates to the manuscript, where applicable.

  

1. We apologize for the lack of quantification and statistics in our results, a point raised by Dr. Artinger and Dr. Wray. To this end, we have now quantified GFP-positive regions in our *in vivo* experiments and report a statistically significant increase in the number of GFP-positive regions in the Cad7 GFP1-10 + N-cad GFP11 electroporated embryos vs. the Cad7 GFP11 + N-cad GFP1-10 electroporated embryos (p = 0.014). We discuss possible reasons for this in the Discussion section, speculating that the reduced number of GFP-positive puncta in the latter could be due to the general larger size of the N-cad GFP1-10 plasmid, potentially precluding robust expression of this construct, and/or intrinsic differences in the ability of neural crest and placode cells to transcribe and translate expression constructs. Although it was not possible to distinguish individual GFP-positive puncta in the cell culture experiments, we have reported the percentage of GFP-positive cells in this assay, along with statistical comparisons. While there is no statistically significant difference in the number of GFP-positive cells after transfection of homophilic split GFP constructs (i.e., Cad7 GFP1-10 + Cad7 GFP11 or N-cad GFP1-10 + N-cad GFP11), we note a statistically significant difference in the number of GFP-positive cells when comparing Cad7 GFP1-10 + N-cad GFP11-transfected cells to Cad7 GFP11 + N-cad GFP1-10-transfected cells, with increased GFP noted in the former (p = 0.013). These findings are in keeping with our *in vivo* results and have been incorporated into the revised manuscript. For each figure, we have also included the number of replicates in the figure legends.
2. We appreciate the feedback on Figure 7 from Dr. Forni and have modified this figure (and the other *in vivo* figures) based on his suggestions. These include bolding the antigen labels and placing them on a different color background, changing the antigen colors in the high magnification images to better visualize different cell populations, adding a lower magnification image of the immunohistochemistry, and including a cartoon diagram to illustrate the location of the image in the context of the developing head.

# Peer review: Reviewer response for version 1

**DOI:** 10.5256/f1000research.134708.r143220

**Published:** 2022-07-18

**Authors:** Kristin Bruk Artinger

**Affiliations:** 1 Department of Craniofacial Biology, University of Colorado Anschutz Medical Campus, Aurora, CO, USA

The manuscript by Halmi *et al* examines interactions between Cad-7 and N-cadherin during assembly of the trigeminal ganglia. Using biochemistry and chick overexpression models, and novel GRASP imaging, the authors that Cadherin-7 and N-cadherin interact to build the ganglia. This paper demonstrates a novel interaction *in vitro* and *in vivo*. It is well written, shows beautiful imaging, and would be of general interest to readers who are interested in cadherins and ganglia assembly. There are some areas of clarification that would strengthen the manuscript. My comments are below:

1. Further clarification to the cell types used is warranted here. It seems the western is done in L cells while staining in Cho cells. Is it because they are not expressed? Is there a way to quantify the levels as compared to endogenous levels in cells that express them?
2. It seems that in, *in vitro* that the Cad7-Cad7 and N-cad-N-cad interaction is much stronger than the Cad-7-N-cad interaction and maybe not as broadly expressed? Here also in Figure 6, the morphology of the cells looks different than in Figure 5. Is this true? Please add this to the results.
3. *In vivo*, the gfp is localized in very few cells. What does the Cad7-Cad7 and N-cad-N-cad interaction look like in the trigeminal ganglia? Is it similarly low or higher? This would add to the data presented here. It also makes me wonder if the interaction changes at different developmental times.
4. In general, quantification of the number of fluorescent puncta would be useful for all the studies but in particular *in vivo.* And does the intensity correlate with the strength of the interaction? If so, these would also be interesting to quantify.

Is the work clearly and accurately presented and does it cite the current literature?

Yes

If applicable, is the statistical analysis and its interpretation appropriate?

Not applicable

Are all the source data underlying the results available to ensure full reproducibility?

Yes

Is the study design appropriate and is the work technically sound?

Yes

Are the conclusions drawn adequately supported by the results?

Yes

Are sufficient details of methods and analysis provided to allow replication by others?

Yes

Reviewer Expertise:

Neural crest and craniofacial developoment

I confirm that I have read this submission and believe that I have an appropriate level of expertise to confirm that it is of an acceptable scientific standard.

# Response

**Published:** 2022-11-29

**Authors:** Lisa Taneyhill

**Affiliations:** University of Maryland, College Park, USA

We thank Dr. Artinger for their thoughtful comments on the manuscript. We have responded to each point below and will make necessary updates to the manuscript, where applicable.

  

1. We apologize for the lack of clarity in the use of two cell lines to examine cadherin expression. Both L cells (from mouse) and CHO cells lack endogenous cadherins (Hong et al., 2010). These cell lines were chosen to allow for ectopic expression of chick cadherins and to prevent any “interference” in the GRASP assay with any other cadherins that might be expressed in them. We initially overexpressed chick N-cadherin in L cells to test and ultimately demonstrate N-cad antibody specificity for immunoprecipitation (old Figure 1), as this experiment takes far less time to perform versus testing antibodies on embryonic tissue. However, we realize that the inclusion of this L cell line data in (old) Figure 1 has served as a source of confusion. To alleviate this, we have now modified the blots in Figure 1 to only include the *in vivo *data from the trigeminal ganglia lysate. The input sample in each of these blots, along with our prior publication (Wu and Taneyhill, 2019), reveals that both the N-cadherin and Cadherin-7 antibodies work by immunoblotting for their respective antigens. Moreover, the IgG control and N-cadherin antibody immunoprecipitation lanes indicate that the N-cadherin antibody is able to immunoprecipitate endogenous N-cadherin in trigeminal ganglia lysates. Given this, we do not think it is necessary to quantify N-cadherin and/or Cadherin-7 expression in other cell lines as our main point was to simply use the L cell transfection system to validate our antibodies. We are sorry again for the confusion this has caused and hope the Reviewer finds this solution acceptable.
2. We thank Dr. Artinger for sharing their insights on the cadherin interactions observed in our GRASP cell culture assays. We agree that the homophilic cadherin interactions appear to be much stronger than the heterophilic cadherin interactions in the *in vitro* GRASP assay, and this is also apparent after quantifying the number of GFP-positive cells (new Table 1). These findings are in agreement with what has been published previously on dissociation constant measurements for homophilic versus heterophilic cadherin interactions in cell aggregates *in vitro* (Katsamba *et al*., 2009). Moreover, N-cadherin homophilic interactions confer a much higher degree of adhesivity than those mediated by Cadherin-7 (Chu *et al*., 2006). After careful review of our GRASP data, we also agree with Dr. Artinger’s assessment regarding the morphology of the cells. Generally, transfection of cells with like cadherins and complementary GRASP constructs gives rise to cells that exhibit a round shape or are only somewhat fibroblastic, with few protrusions emanating from the cell (Figure 5). Conversely, transfection of cells with different cadherins and complementary GRASP constructs causes cells to adopt a much more fibroblastic, and often spindly, morphology (Figure 6). This could be due to the presence of both a Type I (N-cadherin) and Type II (Cadherin-7) cadherin in these cells. A parallel to this can be found *in vivo* with respect to the overlapping expression domains of *N-cadherin* and *Cadherin-7* in the developing chick spinal cord. These neuroepithelial cells are organized in a pseudostratified manner and thus exhibit a spindly morphology as they are densely packed within the neural tube/forming spinal cord (Lin *et al*., 2014). We have now added this text to the Results section and thank Dr. Artinger for sharing these observations, as they have strengthened the manuscript.
3. Dr. Artinger raises an intriguing point about the Cadherin-7-Cadherin-7 and N-cadherin-N-cadherin interactions *in vivo* and how they compare to the GFP puncta (representative of Cadherin-7-N-cadherin interactions) we observe. Unfortunately, we are unable to make exact correlations between cadherin expression and GFP puncta because the presence of GFP relies upon each cell expressing its respective complementary cadherin split GFP construct, but the electroporation itself is mosaic. As such, not every neural crest cell or placode cell will be expressing a cadherin split GFP construct. However, we have now added a new figure (Figure 10) to the manuscript showing endogenous cadherin expression in neural crest cells (Cadherin-7) and placode cells (N-cadherin) in the forming trigeminal ganglion. As evident by these images, Cadherin-7-expressing neural crest cells are positioned next to N-cadherin-expressing placode cells. The majority of interactions at this stage, though, appear between adjacent neural crest cells or placode cells (i.e., cadherin homophilic interactions). These findings are in keeping with the GRASP assay data shown in (new) Figures 7 and 8 and the quantification we have performed (Table 2, see below).
4. We thank Dr. Artinger for this useful suggestion about puncta quantification. We have examined a minimum of four serial sections taken through the forming trigeminal ganglion in at least six embryos after sequential electroporation of respective cadherin split GFP constructs into neural crest cells and then placode cells. Through these analyses, we have now quantified the number of GFP-positive regions observed and report these data in the manuscript. We note a statistically significant increase in the number of GFP-positive regions in the N-cad GFP11 + Cad7 GFP1-10 experiment vs. the N-cad GFP1-10 + Cad7 GFP11 experiment (p = 0.014). Interestingly, we observed similar results in our *in vitro* co-transfection experiments. While we might expect these numbers to be similar, the difference could be explained by the ability of cells generally to effectively express N-cad GFP1-10, or, conversely, the ability of cells to express Cad7 GFP11. We favor the former idea, however, for the following reasons. Given that the *N-cadherin* coding sequence is larger than the *Cadherin-7* coding sequence (~300 nucleotides difference), coupled with the greater size of the split GFP1-10 moiety, it is possible that cells do not readily express N-cad GFP1-10 (compared to N-cad GFP11). Alternatively, perhaps there are inherent differences in the ability of neural crest cells and placode cells to transcribe and translate expression constructs like these, with neural crest cells more easily expressing Cad7 GFP1-10 compared to placode cells expressing N-cad GFP1-10. Finally, the heterophilic interactions we are examining occur in *trans*. As such, they are dependent upon a cadherin split GFP construct getting not only electroporated but also appropriately trafficked, and correctly targeted, to a region of the plasma membrane, where it will then be in close proximity to a complementary split cadherin GFP construct on the other cell type. For these reasons, fewer heterophilic interactions may ensue in a given electroporated tissue.
